# Overgrowth syndromes and pediatric cancers: how many roads lead to *IGF2*?

**DOI:** 10.1101/gad.317792.118

**Published:** 2018-08-01

**Authors:** Ruthrothaselvi Bharathavikru, Nicholas D. Hastie

**Affiliations:** Medical Research Council, Human Genetics Unit, Institute of Genetics and Molecular Medicine, University of Edinburgh, Western General Hospital, Edinburgh EH4 2XU, United Kingdom

**Keywords:** DIS3L2, IGF2, LIN28, Perlman syndrome, Wilms tumor, microRNA, pediatric cancer

## Abstract

Overgrowth syndromes such as Perlman syndrome and associated pediatric cancers, including Wilms tumor, arise through genetic and, in certain instances, also epigenetic changes. In the case of the Beckwith-Wiedemann overgrowth syndrome and in Wilms tumor, increased levels of *IGF2* have been shown to be causally related to the disease manifestation. In the previous issue of *Genes & Development*, Hunter and colleagues (pp. 903–908) investigated the molecular mechanisms by which mutations in the gene encoding the RNA degradation component DIS3L2 lead to Perlman syndrome. By analyzing nephron progenitor cells derived from their newly created *Dis3l2* mutant mouse lines, the investigators showed that DIS3L2 loss of function leads to up-regulation of *IGF2* independently of the let7 microRNA pathway. In a second study in this issue of *Genes & Development*, Chen and colleagues (pp. 996–1007) show that microRNA processing gene mutations in Wilms tumor lead to an increase in the levels of transcription factor pleomorphic adenoma gene 1 (PLAG1) that in turn activates *IGF2* expression. Thus, augmented *IGF2* expression seems to be a common downstream factor in both tissue overgrowth and Wilms tumor through several alternative mechanisms.

## Abstract (precis)

In this Perspective, Bharathavikru and Hastie discuss recent studies published by Hunter et al., investigating the molecular mechanisms by which mutations in the gene encoding the RNA degradation component DIS3L2 lead to Perlman syndrome, and Chen et al., who show that microRNA processing gene mutations in Wilms tumor leads to an increase in the levels of transcription factor PLAG1 that in turn activates IGF2 expression.

Overgrowth syndromes, including Beckwith-Wiedemann syndrome (BWS), Simpson-Golabi-Behmel syndrome (SGBS), and Perlman syndrome, are characterized by macrosomia, facial dysmorphism, intellectual disabilities, and, in some cases, renal abnormalities as well as heart defects (for review, see Kamien et al. 2018). These arise through genetic alterations, including chromosomal translocations, loss of imprinting, duplication, or mutations resulting in altered expression of key developmental genes. Patients with these overgrowth syndromes also have a predisposition to tumors, particularly the pediatric kidney cancer Wilms tumor. However, individuals with BWS and SGBS may also develop other tumors, including hepatoblastoma and neuroblastoma. It is well established that BWS arises through increased expression of the imprinted *IGF2* gene through either paternal *11p15* disomy or loss of imprinting of the maternal allele. Furthermore, increased expression of *IGF2* through these mechanisms is commonly found as a somatic event in Wilms tumors. Work with animal models has provided evidence that increased *IGF2* expression is an important contributing factor in both BWS and Wilms tumor (Caspary et al. 1999; Hu et al. 2011; Huang et al. 2016). Two studies, Chen et al. (2018) in this issue and Hunter et al. (2018) in the previous issue of *Genes & Development*, highlight the significance of *IGF2* up-regulation in the context of Perlman syndrome and Wilms tumor, respectively.

Hunter et al. (2018) addressed the mechanisms underlying Perlman syndrome, which is an autosomal recessive syndrome arising through mutation of *DIS3L2* on chromosome *2q37* (Astuti et al. 2012). DIS3L2 functions as a 3′–5′ exoribonuclease that preferentially degrades oligouridylated RNAs (Lubas et al. 2013). In this context, DIS3L2 has been shown to be the only known exoribonuclease for degrading preprocessed forms of microRNA let7 (Chang et al. 2013). Recent genetic evidence has shown microRNA processing gene (MIRPG) mutations associated with Wilms tumor (Rakheja et al. 2014; Torrezan et al. 2014; Walz et al. 2015; Wegert et al. 2015), characterized by dysregulated microRNA levels. Moreover, microRNA let7 is known to function as a Wilms tumor suppressor in a mouse model through inhibition of the oncogenic target lin28, which is overexpressed in Wilms tumor (Viswanathan et al. 2009; Urbach et al. 2014).

Accordingly, the investigators tested the hypothesis that DIS3L2 loss of function leads to alteration in let7 levels in both human cell lines and mouse model systems. Using genome editing, series of *DIS3L2-*null human cell lines as well as knockout mouse embryonic stem cells were created. In neither case was any change in the expression of the microRNA let7 family members observed. With the help of a CRISPR/Cas9 approach, a *Dis3l2*-null mouse strain was generated by deleting the catalytic domain as well as a strain that models a common exon deletion found in Perlman patients. Further analysis showed that this latter mutation results in a destabilized protein and is functionally null. In the case of both alleles, homozygous mutant animals were perinatal lethal. Embryonic day 18.5 embryos exhibited some of the features of Perlman syndrome, including bradykinesia, abnormal curvature of the spine, and highly penetrant genitourinary (GU) abnormalities. However, there was no overgrowth or evidence of kidney abnormalities or Wilms tumor. Wilms tumors arise from the uninduced metanephric mesenchyme, where the Wilms tumor 1 (*WT1*) gene is expressed. An earlier study showed that overexpression of RNA-binding protein lin28 results in Wilms tumor (Urbach et al. 2014) only if the overexpression was driven using a *Wt1*^*cre*^ line. Hence, to bypass lethality and further investigate the propensity of the *Dis3l2* mutant animals to form Wilms tumors, the investigators crossed a floxed *Dis3l2* line with a *Wt1^cre^* line. The resulting mutant animals in this case survived to adulthood but did not exhibit overgrowth or Wilms tumor, indicating that loss of *Dis3l2* alone is not sufficient for the tumor formation.

To investigate the mechanism, the investigators used state-of-the-art approaches to develop nephron progenitor cells (NPCs) from the mutant mice. As for the cell lines, the *Dis3l2* mutant NPCs did not show any change in mature let7 microRNA levels. However, RNA sequencing analysis of the NPCs revealed a severalfold increase in *Igf2* expression. Hunter et al. (2018) show that the increased *Igf2* level is transcriptional and does not seem to be due to any genetic or epigenetic changes associated with the *H19/Igf2* locus. Given the precedents established in human Wilms tumors and mouse models, this level of up-regulation of *Igf2* would be sufficient to play a causal role in tumorigenesis. However, interestingly, three of the four Wilms tumors that developed in Perlman patients also had loss of imprinting or loss of heterozygosity of *IGF2* (Wegert et al. 2015; Gadd et al. 2017), implicating an additive effect. It is not surprising that Wilms tumors did not develop in the *Dis3l2* mutant mice because it has been shown that, in mouse models, Wilms tumor arises only if *Igf2* up-regulation is on the background of additional mutations, such as in *Wt1* (Hu et al. 2011; Huang et al. 2016). The absence of overgrowth phenotype in these mutants could be because of species- and tissue-specific promoter usage, which needs to be investigated separately. Clearly, as *Igf2* up-regulation is at the transcriptional level, loss of exoribonuclease DIS3L2 must be acting on upstream RNA targets. The transcriptome data on the mutant NPCs should be an informative resource to identify such upstream regulators.

In another interesting study in this issue of *Genes & Development*, Chen et al. (2018) elucidated a regulatory network by which altered microRNA levels may lead to Wilms tumor. Recent studies have shown that in ∼20% of Wilms tumors, mutations of MIRPGs is observed, resulting in altered microRNA levels. Chen et al. (2018) show that *pleomorphic adenoma gene 1* (*PLAG1*) is one of the consistently overexpressed genes in Wilms tumor, where MIRPG mutations are seen. The 3′ untranslated region analysis of *PLAG1* showed microRNA-binding sites for 5p microRNAs, miR34, and miR16, which are down-regulated in Wilms tumor. PLAG1 is a known transcription factor, and one of its important target genes in both disease and development is *IGF2* (Declercq et al. 2008).

In order to understand the role of PLAG1 in kidney development, mouse models were created in which *Plag1* was overexpressed in the developing kidney mesenchyme using *Wt1^cre^* and *Six1^cre^* drivers. In both cases, there was an overexpression of PLAG1 in the kidneys, which resulted in abnormal cysts with neoplastic characteristics. This was associated with up-regulation of *Igf2* in the affected tissue. In a Wilms tumor cell line, the investigators replicated these findings, in which down-regulation of miR16/34 results in up-regulation of PLAG1, which transactivates *Igf2* expression. This increase in *Igf2* leads to activation of the mTORC1 signaling pathway. This is also true in the *Plag1* mouse models, where the cysts show positive expression for PLAG1 and activated mTORC1 pathway, whereas the surrounding kidney cells are devoid of this expression. In agreement with these findings, it appears that *PLAG1* overexpression in Wilms tumors may alternatively arise through genetic, epigenetic, or post-transcriptional mechanisms that most likely manifest as an increase in *Igf2* expression. The investigators also speculate on the possibility of using mTORC1 inhibitors or microRNA-based therapeutic strategies in Wilms tumor based on their observations.

Figure 1 summarizes the different mechanisms that converge on *Igf2* up-regulation, leading to overgrowth and/or Wilms tumor. Unraveling the complex molecular details associated with the overgrowth syndromes and pediatric cancers has been largely possible due to the ability to create mouse models that replicate some of the disease phenotypes. It is now important to determine whether the same mechanisms operate and contribute to the phenotypes in humans. The recent advances in genome editing coupled with embryonic stem/induced pluripotent stem cell-derived organoid cultures should now make this possible.

**Figure 1.**
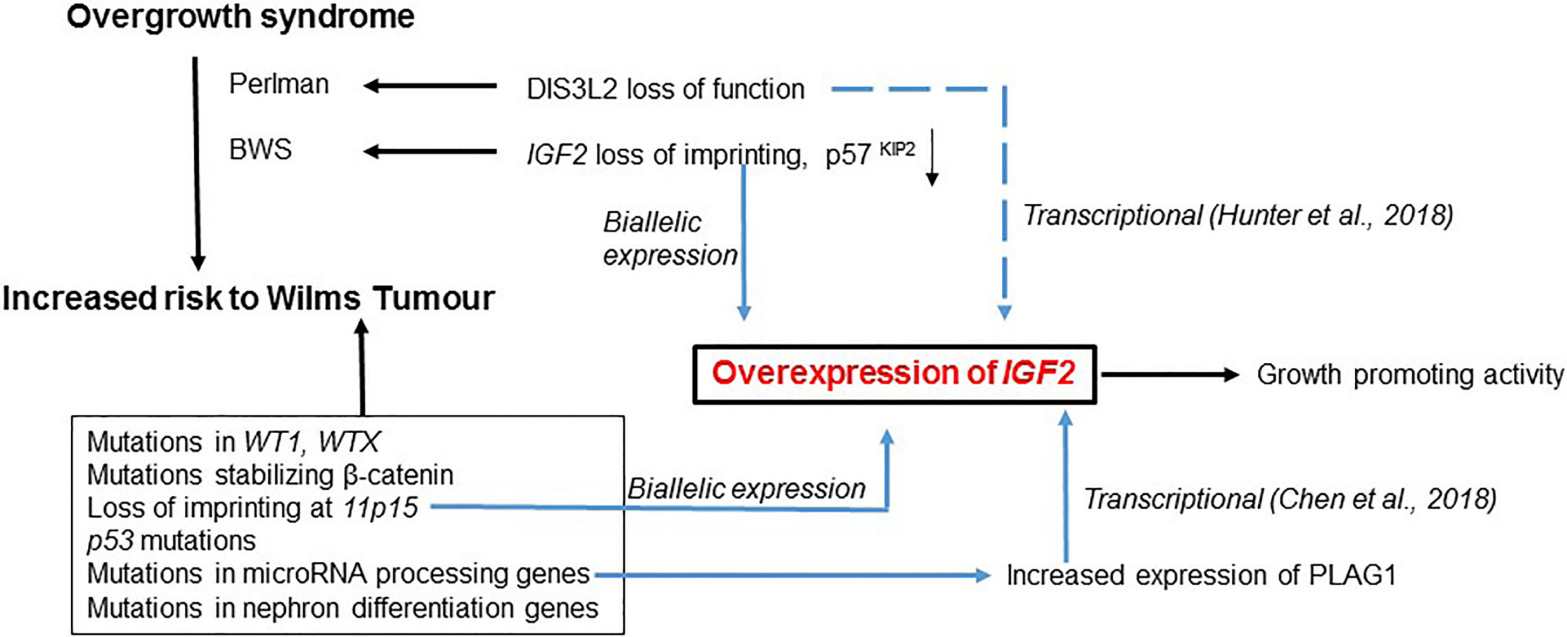
Multiple mechanisms that up-regulate *IGF2* expression in overgrowth syndrome and associated pediatric cancer. The overgrowth syndrome BWS arises due to genetic and epigenetic changes in *IGF2*, whereas a second overgrowth syndrome, Perlman, is caused by mutations in the gene encoding the DIS3L2 exoribonuclease. Loss of DIS3L2 results in an up-regulation of *Igf2.* Patients with Perlman syndrome show an increased predisposition to Wilms tumor. Different categories of mutations have been identified in Wilms tumor. Among these, loss of imprinting at the *11p15* locus results in overexpression of *IGF2*. Mutations in the MIRPGs also lead to an up-regulation of *IGF2* through overexpression of transcription factor PLAG1.
